# Persistent expression of BMP-4 in embryonic chick adrenal cortical cells and its role in chromaffin cell development

**DOI:** 10.1186/1749-8104-3-28

**Published:** 2008-10-22

**Authors:** Katrin Huber, Aylin Franke, Barbara Brühl, Shlomi Krispin, Uwe Ernsberger, Andreas Schober, Oliver von Bohlen und Halbach, Hermann Rohrer, Chaya Kalcheim, Klaus Unsicker

**Affiliations:** 1 Neuroanatomy, Interdisciplinary Center for Neurosciences (IZN), University of Heidelberg, INF 307, D-69120 Heidelberg, Germany; 2 Max-Planck Institute for Brain Research, Deutschordenstr. 46, D-60528 Frankfurt, Germany; 3 Department of Anatomy and Cell Biology, Hebrew University of Jerusalem, Hadassah Medical School, Jerusalem 91120, Israel

## Abstract

**Background:**

Adrenal chromaffin cells and sympathetic neurons both originate from the neural crest, yet signals that trigger chromaffin development remain elusive. Bone morphogenetic proteins (BMPs) emanating from the dorsal aorta are important signals for the induction of a sympathoadrenal catecholaminergic cell fate.

**Results:**

We report here that BMP-4 is also expressed by adrenal cortical cells throughout chick embryonic development, suggesting a putative role in chromaffin cell development. Moreover, bone morphogenetic protein receptor IA is expressed by both cortical and chromaffin cells. Inhibiting BMP-4 with noggin prevents the increase in the number of tyrosine hydroxylase positive cells in adrenal explants without affecting cell proliferation. Hence, adrenal BMP-4 is likely to induce tyrosine hydroxylase in sympathoadrenal progenitors. To investigate whether persistent BMP-4 exposure is able to induce chromaffin traits in sympathetic ganglia, we locally grafted BMP-4 overexpressing cells next to sympathetic ganglia. Embryonic day 8 chick sympathetic ganglia, in addition to principal neurons, contain about 25% chromaffin-like cells. Ectopic BMP-4 did not increase this proportion, yet numbers and sizes of 'chromaffin' granules were significantly increased.

**Conclusion:**

BMP-4 may serve to promote specific chromaffin traits, but is not sufficient to convert sympathetic neurons into a chromaffin phenotype.

## Background

The neural crest (NC) plays a paradigmatic role for studying the diversification of multipotential progenitor cells into distinct cell types. Sympathetic neurons and the endocrine chromaffin cells of the adrenal medulla and extra-adrenal locations are derived from the NC [1]. Both cell types share many characteristics – for example, the synthesizing machinery for noradrenaline (see [2] for a review) – but are very distinct in other aspects. It is widely believed that chromaffin cells and sympathetic neurons develop from the NC via a common sympathaodrenal (SA) progenitor, which has the capacity to give rise to both sympathetic neurons and chromaffin cells. SA progenitors develop in the trunk region near the dorsal aorta [3-6]. In this location they acquire catecholaminergic neuronal features, and then are supposed to re-migrate to the sites of the secondary sympathetic ganglia and the adrenal gland. Chromaffin cell differentiation is believed to involve the inhibition of terminal neuronal differentiation [7], the downregulation of neurofilament (NF), lack of neurites, and the development of large 'chromaffin' dense-core vesicles [2,8-11] However, the differential cues determining either a neuroendocrine or neuronal fate have not been identified as yet. Tissues surrounding NC cells and SA progenitor cells during their migration and at their final locations are considered to be important for the induction of a sympathetic neuronal or chromaffin cell phenotype.

Glucocorticoids secreted by the adrenal cortex have long been thought to be essential for chromaffin cell differentiation [11-14]; however, analysis of glucocorticoid receptor-deficient mice revealed that their adrenal chromaffin cells are largely normal [15]. Other factors provided locally by the adrenal gland, such as transforming growth factor-β, have been shown to be involved in the regulation of chromaffin cell proliferation, but not in chromaffin cell phenotype determination [16].

Bone morphogenetic proteins (BMPs) comprise a family of growth factors that were first identified according to their osteogenic properties [17-19]. Subsequently, they were found to be expressed widely in vertebrate embryonic structures and shown to be involved in a variety of key embryonic processes such as dorsal-ventral axis specification, epithelio-mesenchymal interactions, and apoptosis [20]. BMP-4 and BMP-7 play an important role in the specification of SA progenitors from the NC in the avian embryo [3,4,21] and are expressed in the wall of the dorsal aorta. Overexpression experiments of BMP4/7 and the use of noggin, an inhibitor of BMP-4/7, showed that BMPs are necessary and sufficient for the early induction of a neuronal and catecholaminergic phenotype in NC cells that aggregate in the vicinity of the dorsal aorta [3-5,21].

It has recently been suggested that BMP-4 is required only transiently for an early step of sympathetic neuron differentiation but may block subsequent steps of terminal neuronal differentiation. This hypothesis was based on the observation that NC cells that were treated with BMP-4 form ganglion-like clusters and extend neurites only after withdrawal of BMP-4 [22]. This suggested the possibility that high and maintained BMP expression may result in catecholaminergic cells without neuronal properties, that is, chromaffin cells. We now demonstrate that BMP-4 is expressed in cortical (interrenal) cells of the developing chick adrenal gland but is not detectable in sympathetic ganglia. We provide a detailed analysis of the temporal and spatial pattern of BMP-4 and BMP receptor (BMPR) expression in the embryonic chick adrenal gland. We show that noggin, which inhibits BMP activity, reduces numbers of catecholaminergic cells in explant cultures of the adrenal gland. However, our results from BMP-4 overexpression experiments at the sites of secondary sympathetic ganglia suggest that prolonged exposure of SA cells to BMP-4 promotes the expression of chromaffin traits but is not sufficient to alter the proportion of chromaffin-like cells in the ganglia.

## Materials and methods

### Experimental animals

Fertilized White Leghorn eggs were incubated in a humidified egg chamber at 38°C until embryonic day (E)3, E4, E5, E6, E7 or E9. On the indicated day of incubation, whole embryos were harvested and the stage according to the criteria of Hamburger and Hamilton [23] was determined. Embryos were either fixed in 4% paraformaldehyde overnight or the adrenal anlagen were dissected for tissue cultures or RNA-isolation.

### Tissue culture

Chinese hamster ovary (CHO) cells producing *Xenopus *noggin and dhfr-CHO control cells were a kind gift from Richard Harland and Dale Frank. Cell lines were grown as described previously [24]. Supernatant was collected after a 4-day culture period and was then concentrated 20-fold using a minicon concentrator (CS 15; Millipore, Schwalbach, Germany).

For explant cultures the adrenal anlagen, including the adjacent mesenchyme, were dissected from stage 23 using sharpened insect needles. To prepare collagen gels, 5 μl sodium bicarbonate (5%) was added to 95 μl of rat tail collagen (90%) in DMEM. The collagen solution was put into a 3.5 cm diameter petridish (Costar, Schiphol-Rijk, Netherlands) and adrenal explants were placed on top. After the gel had polymerized, 4 ml of DMEM medium (Invitrogen, Gaithersburg, MD, USA) supplemented with 10% foetal calf serum and antibiotics (penicillin, streptomycin, neomycin (PSN); Invitrogen) were added. The medium contained 1% supernatant of either noggin-producing CHO cells or control dhfr-CHO cells. Explant cultures were incubated in a 95% air/5% CO_2 _atmosphere at 37°C. Every two days 50% of the medium was changed. The explants were fixed after 1, 3 or 5 days in culture and processed for electron microscopy or cryoembedding followed by immunofluorescence staining or *in situ *hybridisation (see below). For 5-bromo-2'-deoxy-uridine (BrdU) labelling and detection, a BrdU-labelling and Detection Kit I (Roche; Mannheim, Germany) was used. The BrdU-labelling solution was prepared according to the manufacturer's instructions in culture medium with and without noggin and added after a culture period of 3 days 1 hour before fixation.

### Histology

To prepare cryosections, paraformaldehyde-fixed tissues were rinsed three times with phosphate buffer and then placed in 30% sucrose in phosphate-buffered saline (PBS) for cryoprotection. Following overnight immersion in sucrose, the tissue was coated with Tissue TEK^® ^O.C.T™ compound (Sakura Finetek Europe B.V, Zoeterwoude, Netherlands, frozen on dry-ice and stored at -70°C until further processing. The tissue was then cut into 12 μm serial sections, mounted on Superfrost™ slides and air-dried for 30 minutes before performing *in situ *hybridisation or immunfluorescence staining.

Non-radioactive *in situ *hybridisation on cryosections and preparation of digoxigenin-labelled probes for chick tyrosine hydroxylase (TH), chick achaete scute-homologue 1 (CASH-1) [25] chick Phox2B [26], chick neurofilament-M [27], chick BMP-4 [28], chick BMPRIA and IB [21], chick steroidogenic factor 1 (SF-1) and chick Sox10 [4] were carried out using a modification of the protocol of D Henrique (IRFDBU, Oxford, UK) as previously described [29]. Chick SF-1 (base-pairs 509–1,288) was cloned by reverse transcription (RT)-PCR using a pGEM-T vector system (Promega, Mannheim, Germany) following the manufacturer's instruction.

For TH immunfluorescence-staining, sections were pretreated with 10% normal rabbit serum in PBS and 0.1% Triton X-100, followed by overnight incubation with polyclonal sheep anti-tyrosine hydroxylase antibody (TH, 1:200; Chemicon International, Temecula, CA, USA) at 4°C. Specimens were rinsed in PBS and incubated with a Cy3™-conjugated rabbit anti-sheep antibody (1:200; Jackson Immunoresearch, West Grove, PA, USA) for 2 h at room temperature. Specimens were then rinsed in PBS, counterstained with 4',6'-diamidino-2-phenylindole dihydrochloride (DAPI; 1:1,000) for 10 minutes, and mounted with Fluorescent Mounting Medium (Dako Hamburg, Germany).

For TH immunohistochemistry slides were pretreated with 3% hydrogen peroxide in PBS for 15 minutes. After incubation with primary antibody as described above, sections were incubated with a biotinylated rabbit anti-sheep antibody (1:200; Vector Laboratories Burlingame, CA, USA), rinsed with PBS and incubated for 1 h with avidin and biotinylated horseradish-peroxidase-macromolecular complex (Vector: Elite ABC reagent) according to the manufacturer's instructions. Sections were then rinsed with PBS and stained with 3-amino-9-ethylcarbazol (AEC; Sigma-Aldrich, Taufkirchen, Germany) according to the manufacturer's instructions. After rinsing with PBS, sections were mounted with Kaiser's glycerol gelatine (Merck, Darmstadt, Germany). HNK-1 (CD57) immunolabelling was performed as previously described [30].

### RNA isolation and RT-PCR

RT-PCR was used to determine the expression of BMP-4 mRNA in adrenal anlagen explant cultures. Total RNA was isolated from tissues using Trizol (Life Technologies, Karlsruhe, Germany) according to the manufacturer's guidelines for extraction of RNA from small amounts of tissue. Before reverse transcription samples were digested with DNase (Roche) for 15 minutes at 37°C followed by inactivation at 70°C for 5 minutes. First-strand cDNA was synthesized in a final volume of 25 μl. Reaction mixtures consisted of 1 μg of total RNA and final concentrations of 1× first strand buffer (1× first-strand buffer (New England Biolabs, Frankurt, Germany): 50 mM Tris-HCl, pH 8.3, 75 mM KCl, 5 mM MgCl_2 _(Biolabs), 10 mM dithiothreitol (DTT)) and 1 mM each of dNTPs (Biolabs), 50 ng/μl oligo-dT primer 18 (Biolabs), 1 U/μl RNase inhibitor (Roche), and 20 U/μl Moloney murine leukemia virus reverse transcriptase (Biolabs). Before adding buffer, dNTPs, and reverse transcriptase, the reaction mixture was heated to 75°C for 10 minutes. After adding the final components, incubation at 37°C for 2 h followed. Finally, the reaction mixture was heated for 10 minutes at 65°C. Negative controls were carried out by omitting the reverse transcriptase.

Following reverse transcription, PCR amplification of the cDNA was carried out using specific primers for chick BMP-4 (5'AGGAGCTTCCACCATGAAGA3' and 5'CGGCTAATCCTGACGTGTTT3'; 413 bp PCR product) and chick GAPDH (5'GTCAACGGATTTGGCCGTAT3' and 5'AATGCCAAAGTTGTCATGGATG3'; 489 bp PCR product). Reactions were performed in an Eppendorf Mastercycler Gradient thermocycler. (Eppendorf, Hamburg, Germany) Reagents were assembled in a final volume of 50 μl with 1 μl of first-strand cDNA, 1 μM forward primer, 1 μM reverse primer, 1× PCR buffer (10× PCR buffer: 200 mM Tris-HCl, pH 9.0, and 500 mM KCl (Promega, Mannheim, Germany), 2.5 mM MgCl_2_, and 0.1 mM each of dNTPs, Taq DNA polymerase (0.5 μl, 2.5 U; Promega) and RNase-free water to 50 μl. cDNAs were amplified for 30 cycles. One round of amplification consisted of 45 s at 94°C, 45 s at 57.3°C, and 1 minute at 72°C. PCR reactions (12.5 μl) were run on agarose gels (Life Technologies, Karlsruhe, Germany) in 1× TAE buffer (0.04 M Tris-acetate and 0.001 M EDTA), and reaction products were visualized after soaking gels in 0.5 μg/ml ethidium bromide solution in distilled water for 10 minutes, with a transilluminator (Intas, Göttingen, Germany). Pictures were taken by a computer-assisted gel documentation system (Intas).

### Overexpression of BMP-4 at the site of developing secondary sympathetic ganglia

Infections of chick embryos with RCAS-BMP-4 viruses and control RCAS viruses were performed as described by Reissmann *et al*. [3]. Embryos were implanted with infected fibroblasts at day 2 at the level of the wing bud, fixed at day 8, and staged as described above. Tissues were kryo-embedded, cut into 12 μm transverse serial sections and then processed for neurofilament-M *in situ *hybridisation followed by TH immunohistochemistry as described above. Numbers of TH-positive/NF-positive and TH-positive/NF-negative cells in sympathetic ganglia were determined in every fifth section. The analyzed region extended from the superior thoracic aperture 1.5 mm into the caudal direction. This region was infected by the RCAS virus in all experimental embryos as shown by *in situ *hybridisation for RCAS-RT.

### Electron microscopy

For electron microscopy, tissue was fixed by immersion in a mixture of glutaraldehyde (1.5%) and paraformaldehyde (1.5%) in phosphate buffer at pH 7.3 for 48 h and rinsed several times with cacodylate buffer (0.1 M). Organs were then post-fixed in 1% OsO_4_/1.5% potassium hexacyanoferrate, rinsed in 0.1 M cacodylate buffer and 0.2 M sodium maleate buffer (pH 6.0) and block-stained with 1% uranyl acetate. Following dehydration through increasing concentrations of ethanol, the tissue was Epon-embedded. Ultrathin sections (50 nm) were examined with a Zeiss EM10.

For counts and measurements of 'chromaffin' granules in secondary sympathetic ganglia, serial ultrathin sections (50 nm) were photographed, digitalized, and stored on a personal computer. The subsequent analysis was performed by an experimenter blinded to the treatment and stage by coding the images. The coded images were analyzed using the software *ImageTool 3.0 *(University of Texas, Health Science Center, San Antonio, USA). The first image that was analyzed was randomly selected (one of the first three images) and starting with that image, every fourth image was analysed.

Two different parameters were analyzed: total numbers of granules within the image; and the mean surface area of large granules. The total numbers of granules were determined by using the 'count and tag' plug-in of *ImageTool*. Total numbers were determined in material derived from stages 31, 32 and 33 (with and without BMP-4 treatment). The data are presented as mean numbers of granules (± standard error of the mean). Using the same images, the mean surface areas of large granule profiles were calculated. Large granules were defined as granules with a profile area bigger than 0.01 μm^2^. Each granule meeting this criterion was measured using *ImageTool*. The data are presented as mean profile area (± standard error of the mean).

### Statistical analysis

For statistical evaluation, a one-way ANOVA, followed by post-hoc test (Newman-Keuls Multiple Comparison test) was performed using GraphPad Prism (GraphPad Software, San Diego, CA, USA).

## Results

### BMP-4 is expressed in adrenal cortical cells during embryonic development

**Figure 1:**
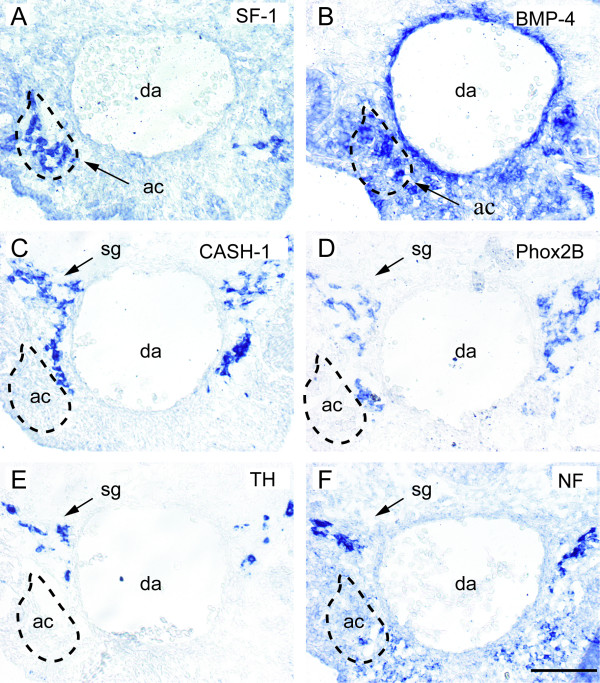
**Transverse sections through a stage 21 (E3.5) chick embryo at the level of the developing adrenal gland**. (A,B) Steroidogenic factor-1 (SF-1) mRNA (A), a marker for adrenal cortical tissue, is expressed at a location corresponding to the site of bone morphogenetic protein-4 (BMP-4) mRNA expression (B). (C-F) A cluster of cells that express the autonomic markers chick achaete-scute homologue (CASH-1, C) and Phox2B (D), but lack tyrosine hydroxylase (TH) (E) and neurofilament (NF) (F), are located in close proximity to the adrenal cortical anlage. Note that cells at a dorsolateral position of the dorsal aorta, corresponding to developing secondary sympathetic ganglia, express TH and NF. ac, adrenal cortical anlagen; da, dorsal aorta; sg, sympathetic ganglion. Bar: 100 μm.

We first conducted an *in situ *hybridisation study to reveal putative sites of BMP-4 mRNA expression in the developing adrenal gland and para-adrenal region. At S21, the earliest stage the adrenal cortex could be identified, BMP-4 mRNA was expressed in the wall of the dorsal aorta and at a site corresponding to the region of the developing adrenal cortex, as identified by expression of the adrenocortical marker SF-1 [31] in adjacent sections (Figure 1A,B). In close apposition to the medial surface of the adrenal anlagen (Figure 1C,D), a group of cells expressing the early autonomic markers CASH-1 and Phox2B [25,32-35] is seen. These cells did not express TH (Figure 1E) or NF (Figure 1F), in contrast to the dorsal cell population, which represents developing sympathetic ganglia (sg). At this early stage of development, cells that express CASH-1, Phox2B, TH, or NF could not be detected inside the adrenal anlagen.

**Figure 2:**
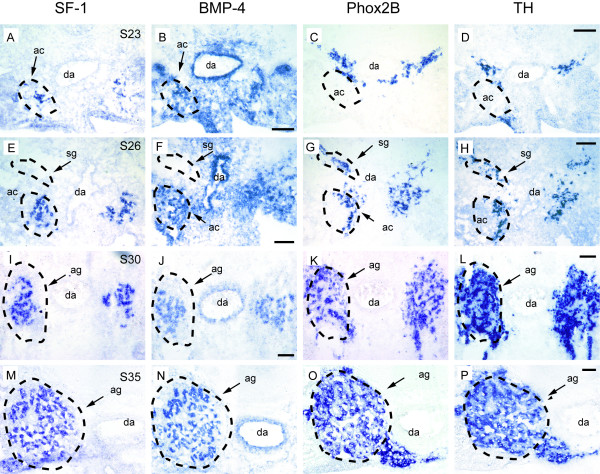
**Expression of bone morphogenetic protein-4 (BMP-4) in adrenal cortical cells at developmental stages S23 (B), S26 (F), S30 (J), and S35 (N)**. Adjacent sections were labelled for steroidogenic factor-1 (SF-1) (A,E,I,M), Phox2B (C,G,K,O) and tyrosine hydroxylase (TH) mRNA (D,H,L,P). The positions of the adrenal cortical anlagen (ac) or the adrenal gland (ag) are marked. (A-D) At S23, the TH/Phox2B-positive chromaffin progenitors are located outside, but closely attached to the adrenal cortical anlage. (E-H) At S26 they have started to invade the adrenal cortical anlage. (I-L) At S30 both cortical and TH-positive chromaffin cells are completely intermingled. da, dorsal aorta; sg, sympathetic ganglion. Bar: 100 μm.

**Figure 3:**
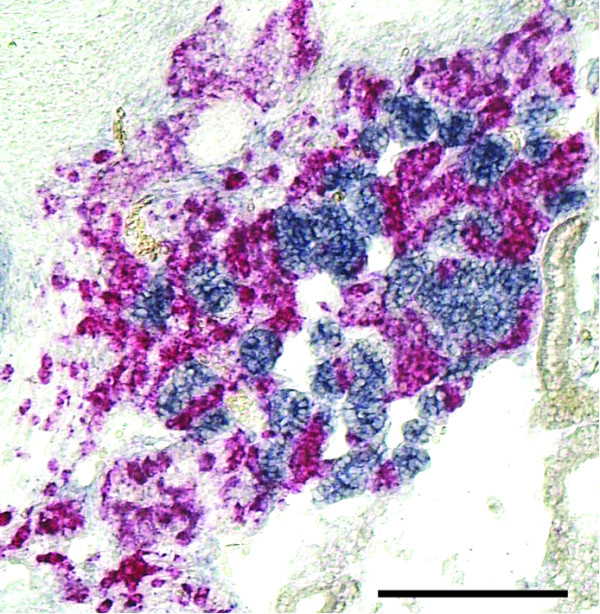
**Double *in situ *hybrization for bone morphogenetic protein-4 (BMP-4; blue) and tyrosine hydroxylase (TH; red) on a section of the adrenal gland of a developmental stage S35 (embryonic day 9) chick embryo**. Note that BMP-4 is expressed in the adrenal cortical (interrenal) cells surrounding the TH-positive adrenal chromaffin cells. Bar: 100 μm.

We next studied adrenal BMP-4 and SF-1 expression at stages 23 through 35 (Figure 2B,F,J,N for BMP-4; Figure 2A,E,I,M for SF-1). Adjacent sections were used for the detection of Phox2B and TH mRNAs (Figure 2C,G,K,O for Phox2B; Figure 2D,H,L,P for TH). At stage 23 (Figure 2C,D), Phox2B and TH mRNA-positive cells were still located outside, but closely apposed to the adrenal anlagen. Starting at stage 26, cells of the adrenal anlage expressing SF-1 and cells expressing Phox2b and TH mRNA became intermingled (Figure 2D,G). BMP-4 was broadly expressed in the mesenchyme ventral to the dorsal aorta, including the area where the adrenal anlage develops. BMP-4 expression became restricted to interrenal cells between stage 26 and 30 and was expressed by adrenal cortical cells until at least E15 (not shown), the oldest developmental age studied. During all stages investigated, BMP-4 expression was also maintained in the wall of the dorsal aorta (Figure 2B,F,J,N) and cardinal veins (not shown), that is, in locations where extra-adrenal chromaffin cells develop. In contrast, sites of the secondary sympathetic ganglia failed to reveal expression of BMP-4 (compare Figure 2F and 2G). Together, these data show that BMP-4 mRNA was expressed early and persisted in those locations where adrenal and extra-adrenal chromaffin cells develop, while BMP-4 mRNA was undetectable at sites of secondary paravertebral sympathetic ganglia. The restriction of BMP-4 expression to interrenal but not chromaffin cells is demonstrated by double labelling for TH mRNA (red) and BMP-4 mRNA (blue) shown in Figure 3.

### BMP-receptors are expressed in chromaffin cells of the developing adrenal gland

**Figure 4:**
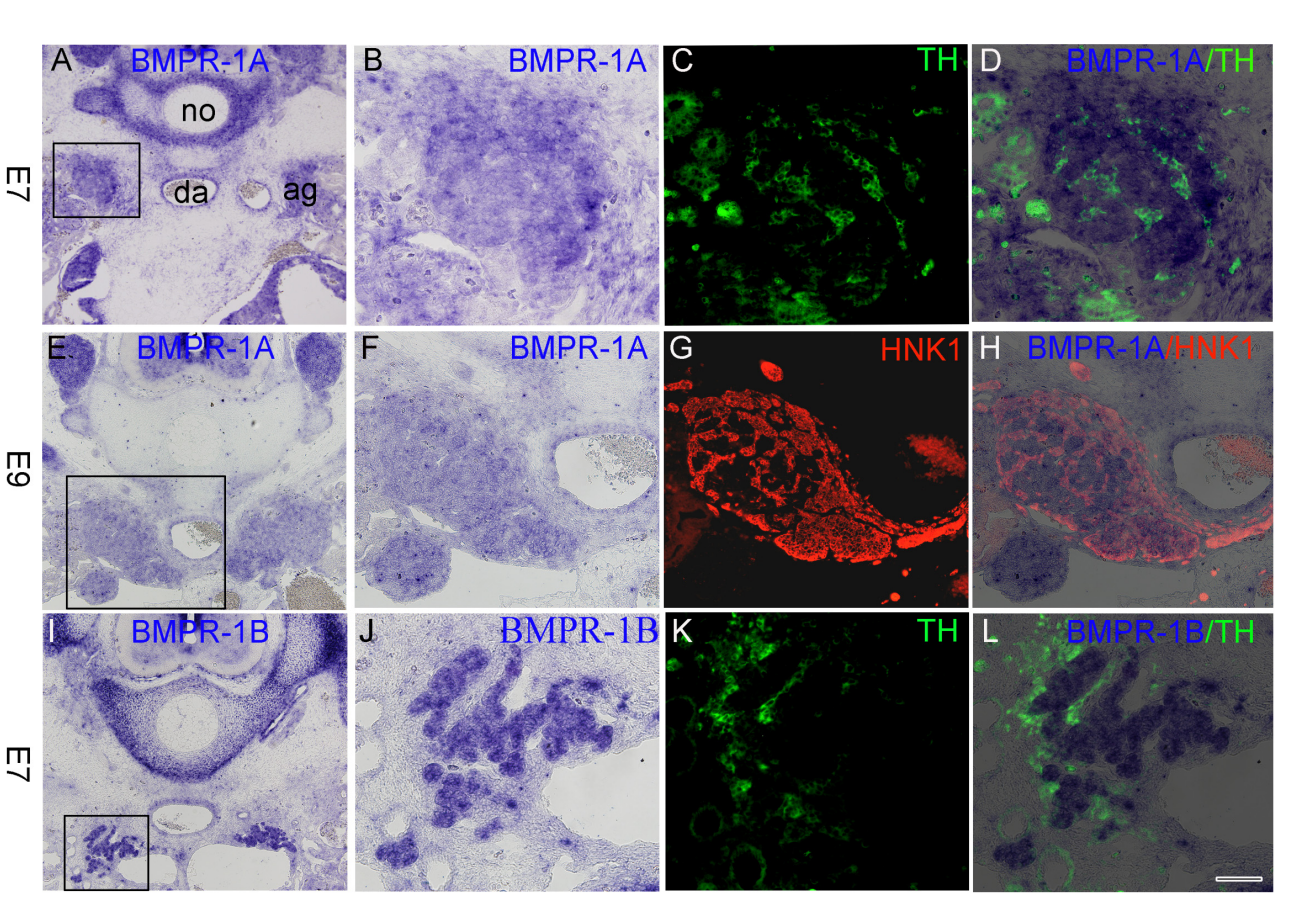
***In situ *hybridisations for bone morphogenetic protein receptor (BMPR)1A (A,B,D,E,F,H) and BMPR1B (I,J,L) in developing chick adrenal glands at embryonic day (E)7 (A-D,I-L) and E9 (E-H)**. Neural crest-derived (chromaffin) cells have been labelled with HNK-1 (CD57, G,H merge) or tyrosine hydroxylase (TH) (C,D merge; K,L merge). Note that BMPR1A is expressed in both adrenal chromaffin and interrenal (cortical) cells (A-H) while BMPR1B is expressed only by interrenal cells (I-L). ag, adrenal gland; da, dorsal aorta; no, notochord. Bar: 175 μm.

The responses to BMP family members are mediated by heterotetrameric complexes composed of type II receptors in concert with type I receptors of either class A or B, which preferentially transduce signalling by BMP2/4 or BMP7, respectively [36]. To begin examining the significance of persistent BMP expression in sites where chromaffin cells develop, we first analyzed the expression of BMPRs 1A and 1B. At E4 and E5, BMPR1A was expressed, *inter alia*, in a few HNK-1-positive sympathoadrenal cells located next to the dorsal aorta (not shown). From E6 till E9 significant expression of receptor transcripts was detected both in TH-positive and in HNK-labelled crest derivatives in the adrenal gland (Figure 4A–H). Additionally, BMPR1A mRNA was also expressed in HNK-negative cells within the gland, the latter probably representing cortical cells (Figure 4H). In contrast, BMPR1B was not found in NC derivatives at any age tested (E4-9), but was strongly expressed in cells with the typical epitheloid morphology of adrenal cortical cells. These results were further confirmed by double staining for receptor types and TH immunoreactivity. At E7, TH-positive chromaffin cells were intermixed among BMPR1B-positive cells but did not produce this receptor (Figure 4I–L). In contrast, both TH-positive chromaffin cells and TH-negative cortical cells were positive for BMPR1A (Figure 4A–D). Hence, both receptor types were expressed in cortical cells of the adrenal gland, but only BMPR1A was synthesized by the chromaffin progenitors.

### Numbers of TH-positive cells in adrenal explant cultures are reduced by noggin-treatment

Expression of BMP-4 in the adrenal cortical anlagen and concomitant expression of BMPR mRNA in adrenal chromaffin progenitor cells suggested a putative function of BMP-4 in chromaffin cell development. Noggin, a secreted inhibitor of BMP-4/7 [37], provides a useful tool for neutralizing BMP-4/7 *in vivo *or *in vitro*, and thereby interfering with BMP signalling. To examine the possible involvement of BMP signalling in chromaffin cell development, explants of the adrenal anlagen from S23 chick embryos were prepared and treated with noggin. Explants contained the cortical area and adjacent TH-positive and TH-negative SA progenitor cells, which had not yet colonized the cortex by the time of excision (Figure 2A–D).

**Figure 5:**
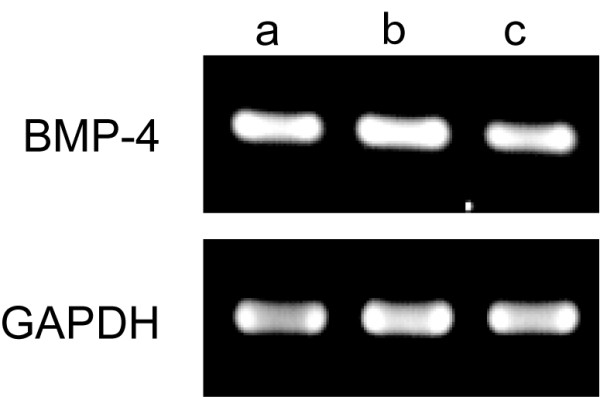
**RT-PCRs for bone morphogenetic protein-4 (BMP-4; upper panel) of adrenal explant cultures of a developmental stage S23 chick embryo after 4 days *in vitro *(lane a)**. Levels of BMP-4 mRNA are comparable to those *in vivo *at S23 (lane b) and S32 (lane c). RT-PCRs of GAPDH (lower panel) were run in parallel.

We first confirmed by RT-PCR that BMP-4 expression is maintained in the adrenal explant cultures. Figure 5 shows that the level of BMP-4 mRNA in the adrenal explants after 4 days *in vitro *is comparable to the amounts of BMP-4 mRNA *in vivo *at the corresponding age.

We next studied the expression of markers specific for adrenal cortical and medullary cells in more detail using antisense and sense riboprobes for SF-1, BMP-4, CASH-1, Phox2B, TH, and NF. All these markers could be detected in the adrenal explants, reflecting a *bona fide in vivo *situation. In addition, cells with the typical ultrastructural features of chromaffin cells and sympathetic neurons could be identified by electron microscopy (not shown).

**Figure 6:**
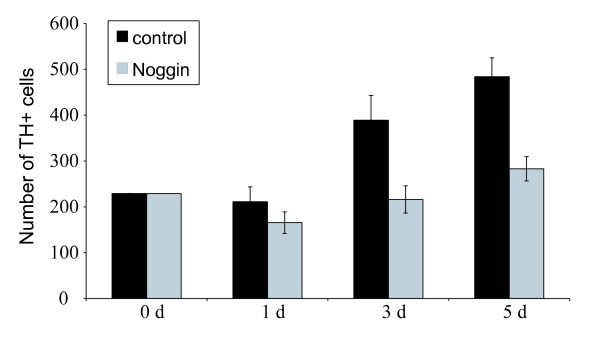
**Quantification of tyrosine hydroxylase (TH)-immunoreactive cells in adrenal explant cultures after 0, 1, 3, and 5 days *in vitro *with or without noggin-treatment**. Numbers of TH-positive cells in explants treated with noggin failed to increase, in contrast to those in control cultures. Counts of TH-immunoreactive cells were performed in every sixth section of the adrenal explant cultures. Data are presented as means ± standard error of the mean. At least seven explants for each group were analyzed.

**Figure 7:**
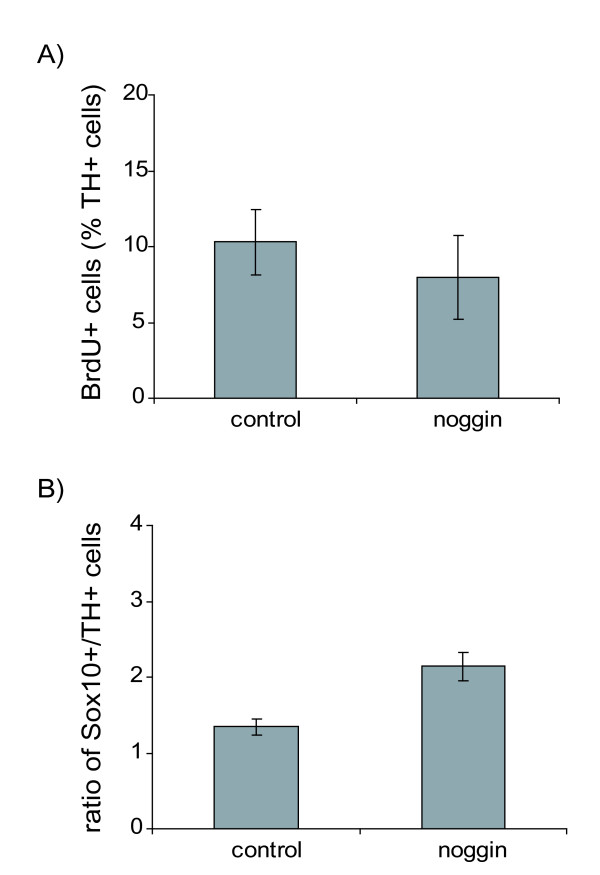
**(A) Numbers of bromo-deoxyuridine (BrdU)-positive cells expressed as a percentage of tyrosine hydroxylase (TH)-positive cell numbers in adrenal explant cultures after 3 days *in vitro*.** Note that there is no significant difference between noggin-treated and control cultures. Data are presented as means ± standard error of the mean. Four explants were analysed per group and every fourth section was counted. (B) Ratio of Sox10-positive/TH-positive cells in adrenal explant cultures after 3 days *in vitro*. Data are presented as means ± standard error of the mean. Five explants were analyzed per group. Sox10 mRNA-positive cells were counted in every eighth section and the adjacent sections were analyzed for TH mRNA-positive cells.

Following the application of noggin for 3 days, no qualitative changes in the expression of these markers or changes in the ultrastructure of cells could be seen. However, noggin treatment prevented the increase in numbers of TH-immunoreactive cells after 3 and 5 days compared to control explants (Figure 6). Together, these data indicate that BMP-4 signalling in the adrenal anlagen affects numbers of TH-positive progenitor cells. BrdU/TH double-labelling experiments did not reveal differences between noggin-treated and control explants (Figure 7A), indicating that noggin does not impair the proliferation of TH positive cells. The ratio of Sox10 mRNA-positive cells to TH mRNA-positive cells was almost twice as high in noggin-treated explants as in control explants (Figure 7B). This suggests that the effect of noggin is unlikely to be due to impairment of proliferation or survival of the Sox10-positive neural crest cells that are the progenitors of the TH-positive SA cells.

### BMP-4 overexpression at the site of developing sympathetic ganglia promotes chromaffin cell differentiation, but is not sufficient to induce a sympathetic neuronal to chromaffin cell shift

**Figure 8:**
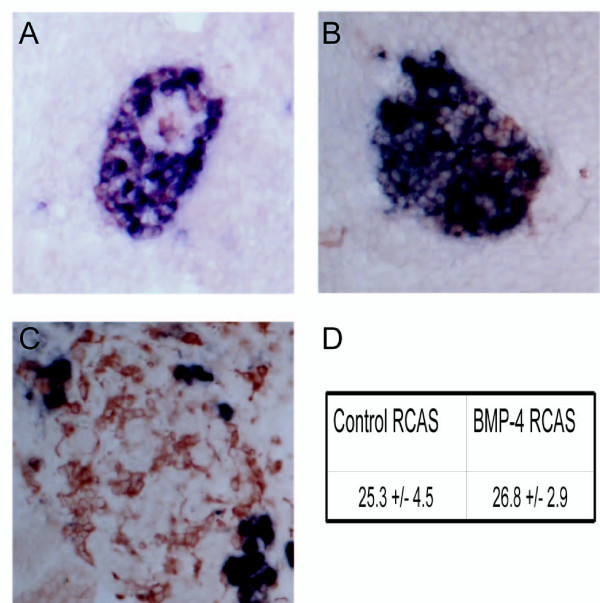
***In situ *hybridisation for neurofilament (blue) combined with tyrosine hydroxylase (TH)-immunocytochemistry (red) on sections of sympathetic ganglia at the upper thoracic level (A,B) and of the adrenal gland (C) of embryonic day (E)8 chick embryos: **(A,C) control embryo; (B) embryo that was transplanted with bone morphogenetic protein-4 (BMP-4)-expressing fibroblasts at E2. Note that the majority of cells in the sympathetic ganglia of both control and BMP-4-overexpressing embryos is neurofilament-positive, while in the adrenal gland most cells are TH-positive, but neurofilament-negative. (D) The percentage of TH-positive neurofilament-negative cells was not altered in BMP-4-overexpressing embryos. Bars: 100 μm.

The continuous expression of BMP-4 in the adrenal cortex and the lack of BMP-4 expression at the site of secondary paravertebral sympathetic ganglia prompted us to investigate whether prolonged expression of BMP-4 is the decisive cue for the differentiation of SA progenitors into chromaffin cells as opposed to sympathetic neurons. To test this hypothesis, we overexpressed BMP-4 by implanting fibroblasts infected with a replication competent RCAS-BMP-4 virus [3]. All infected embryos analyzed at E8 showed massive deformations of vertebral bones and the spinal cord in the infected region, suggesting a *bona fide *effect of overexpressed BMP-4. To analyze a putative shift from a sympathetic neuronal towards a chromaffin cell fate, we determined the numbers of NF-positive/TH-positive cells and NF-negative/TH-positive cells in the paravertebral sympathetic ganglia of the BMP-4 infected region. NF is a marker for sympathetic neurons, but is absent in mature chromaffin cells [38,39] (Figure 8B). However, numbers of NF-negative/TH-positive cells expressed as a percentage of the total number of TH-positive cells were virtually identical in chick embryos treated with BMP-RCAS (26.8 ± 2.9%) and control RCAS virus (25.3 ± 4.5%), respectively. This result indicates that prolonged expression of BMP-4 is not sufficient to induce a chromaffin cell fate in SA progenitors.

#### Ultrastructure of sympathetic ganglia upon BMP-4 overexpression

**Figure 9:**
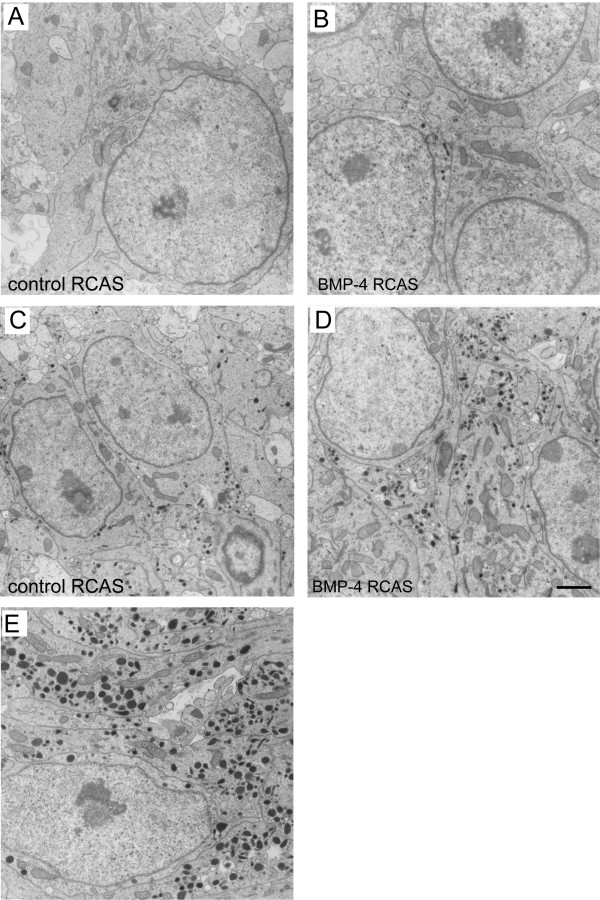
**(A-E) Electron micrographs of sympathetic ganglia at the upper thoracic level (A-D) and of the adrenal gland (E) of embryonic day (E)8 chick embryos: (A,C,E) control embryo; (B,D) embryo that was transplanted with bone morphogenetic protein-4 (BMP-4)-expressing fibroblasts at E2. **Note that two major types of sympathoadrenal cells exist in sympathetic ganglia of both control and BMP-4 overexpressing embryos: cells without or with small granules in the cell body (A,B), and cells with large granules (C,D). Bar: 2 μm.

**Figure 10:**
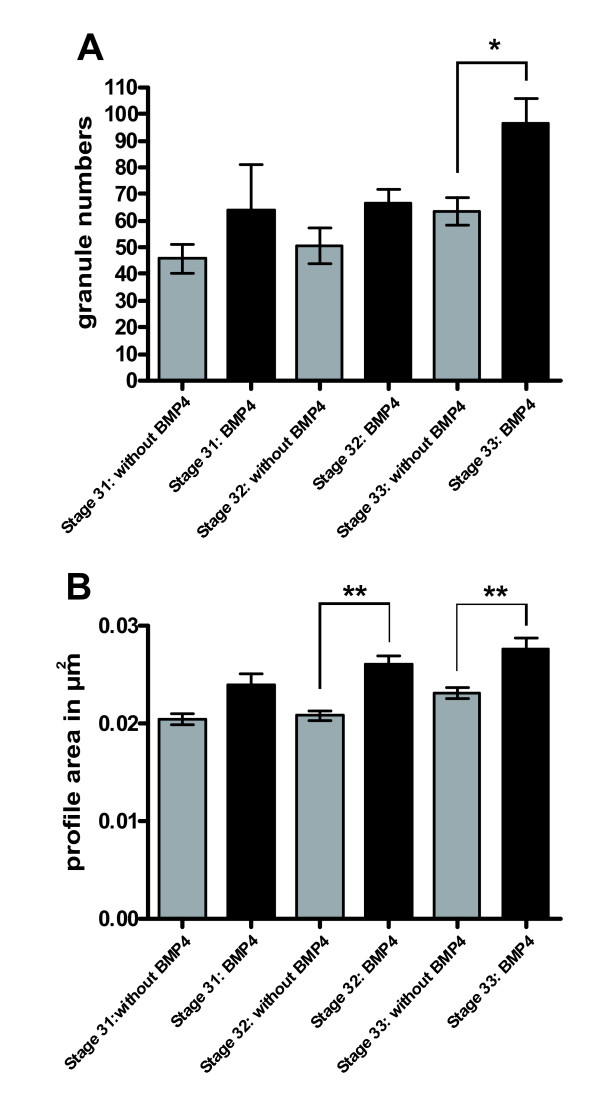
**Numbers and area profiles of 'chromaffin' granules of cells in sympathetic ganglia**. (A) The number of granules in the sympathetic ganglia was significantly enhanced in the group of the bone morphogenetic protein-4 (BMP-4)-treated embryos at stage 33. The data are presented as mean numbers of granules (± standard error of the mean). (B) BMP-4 treatment increased the mean profile area of 'chromaffin' granules in the sympathetic ganglia of stage 32 and 33 embryos. The data are presented as mean profile area (± standard error of the mean).*p ≤ 0.05; **p ≤ 0.01.

To gain further insight into a putative role of BMP signalling in the phenotypic differentiation of chromaffin progenitors, we next analysed the ultrastructure of paravertebral sympathetic ganglia in BMP-RCAS and mock-infected embryos. Chromaffin and chromaffin progenitor cells can be reliably identified by their large (up to 250 nm in diameter) 'chromaffin' granules (Figure 9C–E), which are absent from sympathetic neurons [35] (Figure 9A,B). As shown in Figure 8, cells with large 'chromaffin' granules were found in paravertebral sympathetic ganglia of both BMP-RACS (Figure 9D) and mock-infected (Figure 9C) embryos. Most of these cells were located in the periphery of the ganglia, similar to the location of the TH-positive/NF-negative cells shown in Figure 8A,C. Although these granules were more numerous (Figure 10A) and larger (Figure 10B) in sympathetic ganglia of BMP-treated than in control embryos, the granule-containing 'chromaffin-like' cells in sympathetic ganglia could clearly be distinguished from chromaffin cells in E8 adrenal glands. Figure 9E clearly documents the densities and larger sizes of chromaffin granules in an E8 adrenal gland as compared to 'chromaffin-like' cells in BMP-treated sympathetic ganglia (Figure 8D). Together, both the light microscopic and ultrastructural analyses do suggest that BMP-4 promotes chromaffin differentiation, but is unable to induce a shift of sympathetic neurons into chromaffin cells.

## Discussion

The molecular bases of chromaffin progenitor specification are still enigmatic. A classic model had postulated a common progenitor cell for sympathetic neurons and neuroendocrine chromaffin cells (the SA progenitor), and a crucial role for glucocorticoids in blocking neuronal and promoting neuroendocrine differentiation [7,11,12,40]. Analysis of the glucocorticoid receptor knockout [15,41] failed to support this hypothesis: glucocorticoid receptor-deficient mice had normal numbers of adrenal chromaffin cells, which resembled their wild-type counterparts in virtually all structural and chemical aspects.

In our search for alternative cues, we report in this study that adrenal cortical cells (interrenal cells in the chick) express BMP-4 starting at the beginning of cortical cell assembly. At early stages, BMP-4 mRNA is detected in the wall of the dorsal aorta and in adjacent tissues, particularly those extending ventrally and laterally. These regions include the developing adrenal cortical cells expressing the orphan nuclear receptor SF-1. In addition, they engulf the area lateral to the aorta where NC-derived cells are found and adrenal chromaffin cells differentiate. With ongoing development, BMP-4 expression outside the wall of the aorta becomes restricted to adrenal cortical cells, which by then intermingle with the differentiating adrenal chromaffin cells to form the chick adrenal gland. BMP-4 is continuously expressed by cortical cells at least until E15. Thus, throughout this developmental period, differentiating chromaffin cells are surrounded by cells with high BMP-4 expression. This differs from the situation encountered by the cells destined to form secondary sympathetic ganglia. At their initial differentiation site, the primary sympathetic ganglia, the wall of the dorsal aorta is the major source of BMP-4. On the migration route from primary to secondary sympathetic ganglia, BMP-4 expression is hardly detectable. In secondary sympathetic ganglia, BMP expression is detectable by RT-PCR [42] (K. Tsarovina and H Rohrer, unpublished), yet barely detectable by *in situ *hybridisation (present study; UE unpublished observations). Taken together, BMP-4 expression levels differ dramatically between sites of adrenal gland and secondary sympathetic ganglion formation, provoking the question of whether continuously elevated BMP-4 levels constitute a molecular cue required for chromaffin cell differentiation and counteracting neuronal differentiation.

BMPs have been firmly established in their role in the development of autonomic sympathetic and parasympathetic neurons (see [6,43,44] for reviews). BMPs synthesized by cells in the wall of the dorsal aorta trigger the initial development of NC cells towards noradrenergic sympathetic neurons [3-5]. BMPs induce expression of the transcription factors MASH1, Phox2a/b, HAND2, and Gata2/3 [3,45,46]. Overexpression of BMP-4 [3,22] and BMP depletion by noggin [4] demonstrates that BMPs are sufficient and necessary to induce the expression of the enzymes of noradrenalin biosynthesis, TH and dopamine β-hydroxylase (DBH), and the neuronal markers neurofilament-L, SCG10, neurexin I, and synaptotagmin I in NC-derived precursors. Thus, the available data suggest that BMPs are the decisive stimulus triggering a network of transcription factors necessary for the differentiation of NC cells into noradrenergic sympathetic neurons.

The observation that withdrawal of BMP-4 after a short period in NC cell cultures promotes the formation of ganglion-like aggregates of cells extending neurites and expressing TH, neurexin 1, and synaptotagmin I [22] supports the idea that short exposure to BMP suffices to induce noradrenergic and neuronal differentiation. To test whether prolonged BMP availability suppresses neuronal properties *in vivo*, the effect of virus-mediated overexpression of BMP-4 at sites of sympathetic ganglion formation was analyzed. The expression of the neuronal marker neurofilament-M mRNA in sympathetic neurons, which is barely detectable in chick chromaffin cells throughout embryonic development ([38] and this study), appears unaltered in ganglia at sites of BMP-4 overexpression (this study). The grafted BMP-4-secreting cells were clearly biologically effective, as judged by the massive local overproduction of cartilage, alterations in spinal cord patterning and structure of the dorsal aorta. There are several possible explanations for the failure of BMP-4 to ectopically increase numbers of chromaffin cells, beyond the possibility that high levels of BMP-4 may not specifically induce a chromaffin phenotype. Thus, BMP-4 may not have the capacity to convert committed sympathetic neuronal into chromaffin progenitors, at least not in the given cellular and molecular context. Or chromaffin cells might have been generated, but did not survive.

Even so, BMP-4 distinctly promoted differentiation of chromaffin properties in the sympathetic ganglia, as judged by the increase in the number and size of chromaffin granules. In sympathetic ganglia of chick embryos at E6-9, two cell populations with differently sized granules are present [47,48]. A growing population of cells shows scarce dense core vesicles with approximate diameters of 100 nm while a transient population of cells, which is predominantly located in upper lumbar sympathetic ganglia [48] and was not found in the upper thoracic ganglia analysed in the present study, shows larger granules of up to 300 nm in diameter. It is not clear which of the cell populations is affected by BMP-4 overexpression, and it is currently not clear whether neurofilament-M expression differs between the two populations. One possibility is an effect of BMP-4 on granule size in sympathetic neuron precursors while neurofilament-M expression levels remain refractory. Alternatively, cells with larger granules that may show low neuronal marker expression similar to chromaffin cell precursors may respond with an increase in granule size to BMP treatment. The growing evidence of an early divergence between sympathetic neuron and adrenal chromaffin cell precursors may support the latter scenario.

Several lines of evidence argue for both similarities and differences in the signalling networks involved in the generation of sympathetic neurons and chromaffin cells (see [35] for a review). An initial study analyzing the SA cell lineage in mice lacking MASH1 reported that the neuronal progeny of the SA lineage was largely eliminated, whereas adrenal chromaffin cells were hardly affected [32]. Our re-analysis of the MASH1 knockout revealed that MASH1 was required for orchestrating the normal differentiation program of a majority, but not all, chromaffin cells [49]. Similar to the MASH1 knockout, our analysis of Phox2B-/- mice provided evidence that chromaffin cells are apparently distinctly different from sympathetic neurons in their requirement for Phox2B [50]. Recently, it has been shown that the zinc-finger factor Insm1 (IA1) is expressed early during SA development and that a null mutation of Insm1 affects the development of chromaffin cells and sympathetic neurons differentially [51]. Thus, these analyses of MASH1, Phox2B and Insm1 deficient mice suggest that the SA progenies populating sympathetic ganglia and adrenal glands are not identical with regard to their requirements for these transcription factors. The distinct requirements may reflect distinct origins and distinct characters of SA progenitors, and/or differences in the cellular/molecular environments of sympathetic ganglia and the adrenal gland, respectively. Similar to mice, in the chick embryo differences between sympathetic neuron and adrenal chromaffin precursors have been noted from the earliest stages of differentiation [38]. The presence of neuron-like cells with high neurofilament-L expression levels in adrenal tissue throughout development [38] and the presence of chromaffin-like cells with low neurofilament-M expression in sympathetic ganglia (this study) suggest an early diversification of lineages that cannot be overcome by differences in the environment.

What, then, is the function of BMP-4 in the adrenal gland? Our experiments applying noggin for 3 or 5 days to adrenal explants isolated at S23 strongly suggest that adrenal BMP-4 augments numbers of TH-positive cells, and does so by inducing TH in TH-negative SA progenitors rather than by stimulating proliferation monitored by BrdU incorporation. The notion that adrenal BMP-4 induces TH in SA progenitors that are still TH-negative at the time of their migration into the adrenal anlagen is also supported by our observation that a substantial number of SA progenitors, which are Sox10-, MASH1- and/or Phox2B-positive, but TH-negative, can be found in the vicinity and inside the chick (S23) and E12.5 mouse adrenal anlage [38,45]. Thus, adrenal BMP-4 would serve a similar role as BMP-4 secreted from the dorsal aorta, that is, to induce NC cells to become SA progenitor cells. It is also conceivable that adrenal cortical BMP-4 may act in an autocrine fashion on BMPR-bearing cortical cells in functions that remain to be elucidated.

## Conclusion

Our study has revealed adrenal cortical cells as a site of BMP-4 synthesis. Adrenal BMP-4 probably serves to induce SA-specific transcription factors and TH in cells that colonize the adrenal anlage in a very immature state.

## Abbreviations

BMP: bone morphogenetic protein; BMPR: BMP receptor; BrdU: 5-bromo-2'-deoxy-uridine; CHO: Chinese hamster ovary; E: embryonic day; NC: neural crest; NF: neurofilament; PBS: phosphate-buffered saline; RT: reverse transcription; SA: sympathaodrenal; SF: steroidogenic factor; TH: tyrosine hydroxylase.

## Competing interests

The authors declare that they have no competing interests.

## Authors' contributions

KH participated in the design, the execution and/or the analysis of the majority of the experiments. AF provided and analysed the explant cultures and performed some of the *in situ *hybridisations. SC and CK performed and interpreted the BMPRIA/B *in situ *hybridisations. HR performed the infections of chick embryos with RCAS viruses. UE participated in the execution and interpretation of the *in situ *hybridisations and provided most of the riboprobes. BB prepared electron micrographs and participated in the ultrastructural analysis. AS and OvBH obtained the data on vesicle size and density. KU, KH and CK designed the study, interpreted the results and prepared the manuscript with the help of UE and HR. All authors read and approved the final version of the manuscript.
